# Acute Exacerbation of Interstitial Lung Disease: Early Diagnosis and Treatment

**DOI:** 10.3390/medicina61122097

**Published:** 2025-11-25

**Authors:** Francisco León-Román, Elisa Martínez-Besteiro, David Iturbe, Teresa Peña-Miguel, Marco López-Zubizarreta, Sofía Yerovi-Onofre, Ana María Andrés-Porras, David Jerves-Donoso, Cristina Martín-Carbajo, Carmen López-Represa, Ana Jiménez-Romero, Claudia Valenzuela

**Affiliations:** 1 Pulmonology Department, Hospital Recoletas Salud Campo Grande, 47007 Valladolid, Spain; 2 Medicine and Translational Research Department, Universidad de Barcelona, 08007 Barcelona, Spain; 3 Pulmonology Department, Hospital Universitario de la Princesa, 28006 Madrid, Spain; elisa.martinez.besteiro@gmail.com (E.M.-B.);; 4 Pulmonology Department, Hospital Marqués de Valdecilla, 39008 Santander, Spain; 5 Pulmonology Department, Hospital Universitario de Burgos, 09006 Burgos, Spain; 6 Pulmonology Department, Complejo Asistencial de Ávila, 05004 Ávila, Spain; 7 Pulmonology Department, Hospital General de Segovia, 40002 Segovia, Spain; 8 Pulmonology Department, Complejo Asistencial Universitario de Palencia, 34005 Palencia, Spain; 9 Pulmonology Department, Complejo Asistencial de Soria, 42005 Soria, Spain; 10 Pulmonology Department, Complejo Asistencial de Zamora, 49022 Zamora, Spain; 11 Pulmonology Department, Hospital Universitario Río Hortega, 47012 Valladolid, Spain; 12 Pulmonology Department, Hospital El Bierzo, 24411 Ponferrada, Spain

**Keywords:** exacerbation, interstitial lung disease, diagnosis, treatment

## Abstract

Diagnosis and treatment of acute exacerbation of interstitial lung disease (AE-ILD) continue to be challenging. The annual incidence of AE in idiopathic pulmonary fibrosis (IPF) is 5% to 15%, with an in-hospital mortality exceeding 50%. Similar annual incidence and mortality rates have been documented in other ILDs. The pathogenic mechanisms underlying AE are not entirely clear, although they could involve an acute injury or inflammatory process in previously affected lung tissue, with histological features of diffuse alveolar damage, similar to acute respiratory distress syndrome. AE-ILD is defined based on the following criteria: acute respiratory worsening within 30 days in a patient with a previous or concurrent diagnosis of ILD accompanied by new bilateral ground-glass abnormalities and/or consolidation on high-resolution computed tomography after ruling out heart failure or fluid overload. Pharmacologic treatments such as corticosteroids, antibiotics, and immunosuppressants have been and continue to be used despite scarce evidence from randomized placebo-controlled clinical trials. Oxygen therapy and ventilatory support are key elements of treatment of AE-ILD. The aim of our article is to provide an updated review on the diagnosis and treatment of AE-ILD and to propose practical algorithms for management.

## 1. Introduction

Interstitial lung disease (ILD) comprises a heterogeneous group of conditions characterized by an inflammatory and/or fibrosing response of the interstitial space. Idiopathic pulmonary fibrosis (IPF) is the prototypical progressive and chronic fibrosing ILD, and while antifibrotic agents can slow the loss of lung function, mortality associated with late diagnosis and lack of curative treatments for IPF remains relevant [1]. The progress of ILD varies over time, with some patients remaining stable and others experiencing slow, gradual loss of lung function or progressive forms similar to IPF, accompanied by worsening symptoms, lung function, and quality of life; eventually leading to respiratory failure and considerable morbidity and mortality [2]. Acute exacerbation (AE) is a severe complication in established ILD. It consists of acute or subacute worsening of lung function associated with an unfavorable prognosis [3]. AE has been characterized mainly in patients with IPF. However, data on AE are increasingly being reported in other forms of ILD, such as ILD associated with connective tissue disease (CTD-ILD), hypersensitivity pneumonitis, and drug-induced ILD, all of which share similar clinical features [4,5,6]. The initial definition of AE was proposed in 2007. The condition comprised acute respiratory worsening with onset within 30 days in a patient previously diagnosed with IPF accompanied by new bilateral ground-glass opacities and/or consolidation on chest high-resolution computed tomography (HRCT) scan. Furthermore, it was important to rule out causes such as active identifiable infection (using endotracheal aspirate or bronchoalveolar lavage [BAL]), heart failure, pulmonary thromboembolism, or any other obvious cause accounting for impaired lung function [7]. The term “suspected AE-IPF” was used when the proposed diagnostic criteria were not met [7]. This definition was revised and updated in 2016 by an international expert panel, which proposed 4 criteria: acute worsening of lung function within 30 days in a patient with a previous or concurrent diagnosis of IPF, accompanied by new bilateral ground-glass abnormalities and/or consolidation on the chest HRCT scan not completely explained by heart failure or fluid overload, thus eliminating the need to rule out infection stipulated in the 2007 criteria [3,7]. Moreover, the authors established that AE-IPF could be “idiopathic” (with no identified trigger) or “triggered” by events such as infection, aspiration, and surgery [3]. This change was based on better knowledge of the underlying pathophysiological mechanisms and risk factors, thus enabling better clinical application of the diagnostic criteria. The pathogenic mechanisms underlying AE are not completely clear, although, in principle, they involve an acute lesional or inflammatory process on previously affected lung tissue, with histologic characteristics of diffuse alveolar damage, similar to acute respiratory distress syndrome [3,4,7]. The various factors that can act as triggers include respiratory infection, surgical procedures, exposure to toxins, and gastroesophageal reflux [3]. The annual incidence of AE-IPF ranges from 5% to 15%, with an in-hospital mortality rate exceeding 50% and rising to over 90% in cases requiring invasive mechanical ventilation (IMV). Similar frequencies have been reported for other ILDs, although with a lower level of evidence, and with an equally severe impact on survival. Prognosis is further worsened by several risk factors, including advanced ILD, a history of prior exacerbation, comorbidities, and active smoking [4,8,9,10]. The aim of our article is to provide an updated review on the diagnosis and treatment of AE-ILD and to propose practical algorithms for its management.

## 2. Materials and Methods

The present article was designed as a narrative review of the available evidence on AE-ILD. The aim of our study was to synthesize up-to-date information regarding its definition, etiopathogenesis, diagnosis, and treatment, as well as to propose diagnostic and management algorithms for AE-ILD. The authors acknowledge the need for future validation of these algorithms, while also advocating for their simplicity and applicability in routine clinical practice. The literature search was conducted using databases such as PubMed, Google Scholar, and Scopus, including information available up to October 2025. Keywords used for the search included “interstitial lung disease,” “acute exacerbation,” “acute respiratory deterioration,” “risk factors,” and their Spanish equivalents. Owing to the limited evidence available on this condition, the review incorporates original articles, review papers, clinical practice guidelines, observational studies, case series, and case reports. No strict methodological design criteria were applied due to the narrative nature of the review, although clinical relevance and recent contributions to the diagnosis and treatment of AE-ILD were prioritized. Pediatric studies and articles without full-text access were excluded. The information gathered is presented below in different thematic categories to facilitate a comprehensive interpretation of the results.

## 3. Summary of Findings and Discussion

### 3.1. Diagnosis

**Figure 1:**
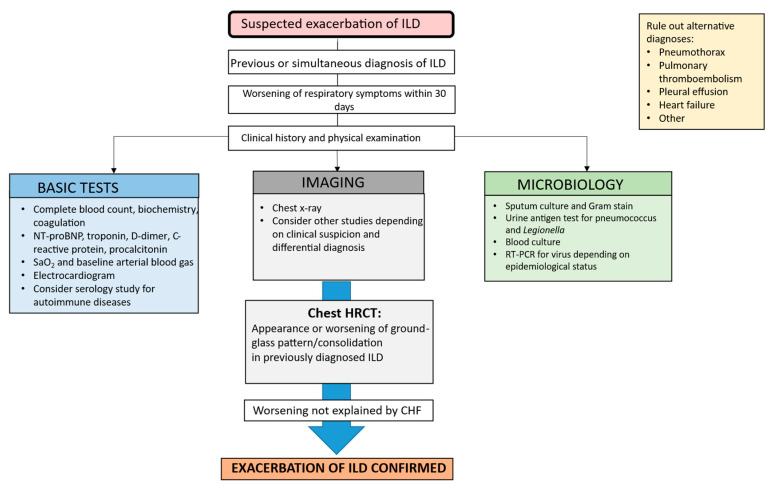
Proposed algorithm for diagnosis of acute exacerbation of interstitial lung disease. ILD: interstitial lung disease; SaO_2_: arterial oxygen saturation; RT-PCR: reverse transcriptase polymerase chain reaction; HRCT: high-resolution computed tomography; CHF: congestive heart failure.

Clinically, AE manifests as respiratory worsening with increased dyspnea and reduced oxygen saturation over a short period (usually within 30 days) in patients previously diagnosed with ILD. Increased cough may also be present, but fever is uncommon. Therefore, its presence should indicate the need to actively investigate infection. In patients with CTD-ILD, respiratory worsening can co-occur with systemic symptoms (arthralgia, myalgia, rash), thus hampering the differential diagnosis [4,8]. Diagnostic criteria have been established for AE-IPF, and while there are no specific criteria for AE in other ILDs, those established for IPF are generally extrapolated. Similarly, there are no international guidelines for the diagnosis and management of AE-IPF or other ILDs. Therefore, diagnostic algorithms capable of appropriately identifying AE must be designed. Inclusion of clinical, radiological, and microbiological variables, as well as serological markers, could prove useful, both for diagnosis and for early management of this condition, whose prognosis continues to be poor. Consequently, the authors of this review have developed a diagnostic algorithm that integrates clinical evaluation, blood biomarkers, microbiological tests, and radiological features to ensure a rigorous differential diagnosis. This approach enables the reliable exclusion of heart failure and opportunistic infections—such as *Pneumocystis jirovecii* pneumonia—in immunocompromised patients, among other entities, while ensuring compliance with all established diagnostic criteria for AE-ILD (Figure 1) [5].

#### 3.1.1. Laboratory Tests

There are no specific diagnostic laboratory tests for AE, with most approaches aimed at making the differential diagnosis and identifying potential triggers. Basic laboratory tests should include biochemistry and a complete blood count, which are useful for identifying infection-related findings, such as leukocytosis and kidney or liver alterations, and for ruling out acute anemia. Arterial blood gas analysis confirms new-onset hypoxemia or worsening of established hypoxemia in patients with more advanced ILD and the potential need for oxygen therapy. Other important laboratory tests in the differential diagnosis include B-type natriuretic peptide, troponins, and D-dimer [11]. Procalcitonin is not indicative of ILD, and its role in AE is controversial, although it is well recognized as a marker of infection. High levels of procalcitonin have been associated with bacterial coinfection, and while procalcitonin is not specific and its usefulness as an isolated biomarker is limited, it can prove helpful when combined with other clinical and microbiological findings for differentiating between idiopathic AE and AE secondary to acute respiratory infection, thus reducing unwarranted use of antibiotics and its associated risks [12]. As for the autoimmune profile, no studies have systematically evaluated whether this varies during an AE compared with previous values or whether it has relevant clinical implications. The prevalence of serology data for autoimmunity in patients with ILD ranges from 60% to 70% in the early diagnostic work-up, with positive autoantibody values, even in the absence of systemic symptoms [6]. However, there is no evidence that repeating autoimmunity testing during an AE adds diagnostic or prognostic value. Therefore, in patients already diagnosed with ILD and a previously negative autoimmune profile, routine repeat serology testing during the AE is not warranted, except in the case of new-onset systemic symptoms raising suspicion of CTD-ILD. In any case, when a patient has recent-onset ILD or unexplained AE, a full autoimmune panel should be run or repeated as part of a new diagnostic work-up, since there could be implications for the management of cases requiring specific immunosuppression [6].

#### 3.1.2. Microbiology

Infection can be reasonably ruled out using basic microbiology tests such as sputum culture, urine antigen testing for *Legionella* and pneumococcus, and blood culture, which is particularly indicated in the case of fever or other findings that might lead us to suspect infection. Nasopharyngeal exudate is recommended for detection of respiratory viruses as possible triggers of AE [4,7]. BAL is no longer a necessary test for ruling out AE. Removal of BAL from the 2016 document was a positive change in daily clinical practice, since its use in AE was limited by severe respiratory insufficiency, which impeded its performance. Therefore, it is reserved for very specific cases of AE with an unclear diagnosis or suspicion of an opportunistic infection, especially in immunosuppressed patients [5].

#### 3.1.3. Imaging Tests

Chest x-ray is useful as an initial test, although its sensitivity is low. It usually reveals bilateral ground-glass opacities with a variable distribution that are often diffuse and superimposed on the chronic changes typical of underlying ILD [4]. Chest HRCT is the most sensitive and specific test in the diagnosis of AE. The characteristic findings include new ground-glass opacities or consolidation on a background of previous interstitial involvement, with no evidence of fluid overload or significant pleural effusion [3,8]. The distribution may be diffuse, peripheral, or basal, and it does not always spare areas previously affected by fibrosis. Pulmonary ultrasound, a noninvasive technique that is increasingly used in respiratory disease, can be useful in the differential diagnosis of AE and other conditions, such as acute pulmonary edema, pleural effusion, and pneumothorax [13].

#### 3.1.4. Electrocardiography and Echocardiography

Both tests can rule out cardiogenic causes. Electrocardiography is used to rule out arrhythmia, ischemia, and indirect signs of left ventricular overload. Transthoracic echocardiography makes it possible to evaluate ventricular function and signs of pulmonary hypertension and rule out heart failure as a cause of respiratory worsening [3,5].

### 3.2. Role of Immunological and Molecular Biomarkers

In recent years, biomarkers such as serum Krebs von den Lungen-6 (KL-6), surfactant protein D (SP-D), and procalcitonin have been used for the identification and prognostic evaluation of AE [14]. KL-6 is a high-molecular-weight protein regulated by the *MUC1* gene and expressed mainly by type II alveolar epithelial cells and activated bronchial epithelial cells in response to damage and regeneration of the alveolar epithelia. KL-6 is the most relevant and most widely studied biomarker in ILD to date owing to its role in prognosis: high KL-6 levels are associated with more severe and progressive ILD, with a risk of AE and hospitalization, and greater mortality [15]. SP-D is a glycoprotein that is synthesized and secreted mainly by type II alveolar epithelial cells. It has both antimicrobial and immunomodulatory functions. A recent meta-analysis showed its serum levels to be associated with progression, AE, and mortality in ILD [16]. As for D-dimer, a retrospective study of a mixed population of patients with ILD found that high D-dimer levels were associated with a 10-fold greater risk of developing AE during the 3 months following its determination [17]. Telomere shortening, matrix metalloproteinases, mucin 5B (MUC5B), human epididymis protein 4 (HE4), periostin, osteopontin, and lung microbiome, among others, have been associated with AE-ILD and identified as independent mortality factors in several studies, although the authors advocate for more rigorous scientific research [14]. Lastly, other potential biomarkers studied in AE-ILD include the following: the neutrophil/lymphocyte ratio, which seems to be associated with lower survival in AE-IPF and AE associated with systemic sclerosis and dermatomyositis/polymyositis; interleukin (IL) 6, which has been reported to be a possible independent risk factor for AE when found at high levels (both in IPF and in non-IPF ILD); and cell populations in BAL fluid (lymphocytosis has been associated with a more favorable prognosis, whereas neutrophilia is associated with poorer prognosis in AE-ILD) [14]. The authors of this review consider the potential importance of biomarkers in the diagnosis, prognosis, and treatment of AE-ILD. However, several relevant factors limit their use in routine clinical practice, including cost and availability, the lack of universal and standardized cutoff values, and finally, the scarcity of scientifically rigorous studies aimed at improving their specificity and clinical applicability.

### 3.3. Management of AE in IPF and Other ILDs

**Figure 2:**
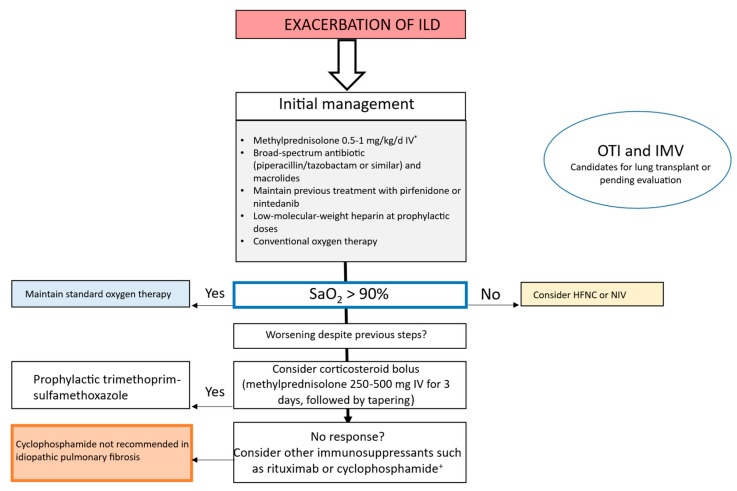
Proposed algorithm for the management of acute exacerbation of interstitial lung disease. ILD: interstitial lung disease; IV: intravenous; SaO_2_: arterial oxygen saturation; HFNC: high-flow nasal cannula; NIV: noninvasive ventilation; OTI: orotracheal intubation; IMV: invasive mechanical ventilation. * No current evidence on the dose or duration of corticosteroids. ^+^ Very limited evidence.

There are no clinical practice guidelines for management of AE-ILD. Despite scant supporting evidence, management of AE-ILD is currently based on corticosteroids, immunosuppressants, broad-spectrum antibiotics, and both invasive and noninvasive ventilation [18,19]. Lung transplantation could be an alternative when indicated and after a rigorous assessment, with no effect on survival or increase in complications compared to patients who undergo transplantation with stable ILD [20,21]. Nevertheless, given the different types of ILD, management should be tailored both to the patient and to the specific condition. Considering the previously mentioned limitations, the authors of this article have developed a treatment proposal for AE-ILD based on the currently available evidence. This approach includes corticosteroids, broad-spectrum antibiotics, and oxygen therapy as first line management. High-dose corticosteroids and immunosuppressive agents are reserved for cases that fail to show an adequate response to the preceding measures, along with consideration of adding *Pneumocystis jirovecii* prophylaxis (Figure 2).

### 3.4. Ventilatory Support in AE-ILD

International guidelines on management of IPF make a weak recommendation against IMV for treatment of respiratory failure in IPF owing to the high associated mortality in this setting [3]. However, IMV can be considered an alternative until a response to established treatment is obtained in specific cases and as a bridge to lung transplantation in combination with extracorporeal membrane oxygenation [22,23]. High-flow nasal cannula (HFNC) and noninvasive ventilation (NIV) are components of noninvasive ventilatory support (NIVS), which is generally used for treatment of respiratory failure in AE-ILD and as a palliative strategy for control of dyspnea [24]. Given the limited information on NIVS in AE-ILD, HFNC are usually considered the approach of choice because they are better tolerated, control dyspnea and respiratory secretions appropriately, and enable oral feeding and communication with the patient [25]. Moreover, NIV has a similar effect to that of HFNC with respect to improving oxygenation and controlling respiratory effort, although in advanced fibrotic ILDs, structural rigidity and the risk of barotrauma reduce any potential beneficial effect [25,26].

### 3.5. Pharmacologic Treatment of AE-ILD

#### 3.5.1. Corticosteroids

No clinical trials to date support the use of corticosteroids or specific doses in AE-ILD. A recent meta-analysis showed that administering these agents during AE-IPF was associated with increased mortality [27]. In a systematic review published in 2025, prednisolone at >1 mg/kg reduced 90-day mortality in patients with non-IPF AE-ILD, as well as in-hospital mortality associated with early tapering of corticosteroids. Results were inconsistent for IPF [28]. Nevertheless, clinical practice guidelines make a weak recommendation in favor of corticosteroids in AE-ILD [3]. Similarly, diffuse alveolar damage, organizing pneumonia, and other inflammatory patterns have been reported as histopathology findings in AE-ILD (in both IPF and in other conditions) and usually respond to corticosteroids [29]. Therefore, until studies with sufficient scientific rigor are available, the authors of this review recommend initiating methylprednisolone or equivalent at doses of 0.5–1.0 mg/kg/day, reserving higher doses of 250–500 mg/day for 3 days followed by a tapering regimen for refractory cases.

#### 3.5.2. Antibiotics

The lung microbiome may be affected by the microbiome of the intestine and oropharynx, thus increasing bacterial load in ILD and associated mortality [30]. In patients with IPF, a significant increase in bacterial load associated with *Campylobacter* and *Stenotrophomonas* during AE has been reported, as has a possible negative impact of anaerobic bacteria and viruses [31]. Therefore, therapy should be based on broad-spectrum antibiotics that cover typical and atypical bacteria during AE-ILD, without forgetting viral and fungal infections and those caused by *Pneumocystis jiroveci* in suspected cases [32]. As for combination of a broad-spectrum antibiotic with azithromycin, a 2017 study showed that when administered at 500 mg/d for 5 days, azithromycin significantly reduced mortality compared with quinolones in patients with AE-IPF [33]. These findings could be explained by the immunomodulatory and anti-inflammatory activity of macrolides, a probable antifibrotic effect, reduced frequency of exacerbations, and control of respiratory secretions [34]. Therefore, broad-spectrum antibiotics such as meropenem or piperacillin-tazobactam combined with macrolides could be used initially until the results of a targeted microbiology study are available.

#### 3.5.3. Immunosuppressants

Few data are available on the treatment of AE-ILD with immunosuppressants, and most studies are retrospective and case reports. The EXAFIP study was a double-blind randomized clinical trial in which patients with AE-IPF received high-dose corticosteroids and intravenous cyclophosphamide or placebo. The authors showed that mortality was increased at 3 months in patients with AE-IPF in whom intravenous cyclophosphamide pulses were added to corticosteroids [35]. These findings constitute evidence against using cyclophosphamide in AE-IPF. However, some studies present data in favor of and against cyclophosphamide in non-IPF AE-ILD, especially in CTD-ILD. Consequently, such controversial evidence has led the authors to recommend administering this drug on an individual basis [36,37]. Furthermore, rituximab combined with plasmapheresis and immunoglobulins has been shown to improve oxygenation and effort tolerance in patients with AE-IPF; a multicenter clinical trial is currently underway to evaluate the effect of this triple therapy in patients with AE-IPF [38,39]. Moreover, evidence from case reports has shown rituximab to be effective in AE-ILD that is refractory to standard treatment in patients with conditions such as rheumatoid arthritis, scleroderma, Sjögren syndrome, antisynthetase syndrome, hypersensitivity pneumonia, organizing pneumonia, and lymphocytic interstitial pneumonia [40,41,42,43]. A 2025 retrospective study showed that baricitinib improved 90-day survival in patients with idiopathic AE-ILD, although the results should be confirmed in clinical trials [44]. Positive results have been reported for tacrolimus, cyclosporine A, tofacitinib, and other immunosuppressants administered to treat AE-ILD, although findings are limited by sample size [45,46,47]. Consequently, the authors of this review recommend the use of immunosuppressive drugs in individualized cases of AE-ILD that are refractory (progressive worsening of respiratoy status with incremental need of respiratory support) to the previously described treatments.

#### 3.5.4. Other Treatments

Polymyxin B hemoperfusion is a safe treatment strategy that improves oxygenation and prognosis in patients with both IPF and non-IPF AE, although data are limited [48,49]. A meta-analysis published in 2020 found that treatment with recombinant soluble human thrombomodulin reduced all-cause mortality at 3 months in AE-IPF, with no significant adverse events. Another study showed the same effect in idiopathic AE-ILD, although the authors recommend validating their data in a randomized clinical trial [50,51]. Furthermore, antifibrotic treatment has been shown to reduce the risk of death and AE in IPF [52]. Antifibrotic agents reduce disease progression and mortality in other non-IPF fibrotic ILDs. In a 2025 study, nintedanib was found to reduce the risk of AE, hospital stay, and mortality in AE-ILD. In the case of pirfenidone, currently available data are inconclusive [53,54]. No clear evidence on when to start antifibrotic treatment in AE-ILD is available, although it would be advisable to maintain the antifibrotic drug if it is being administered before the AE or during a stable phase of AE, providing the criteria for use are met [55,56]. Patients with ILD are at an increased risk of venous thromboembolism. Therefore, during AE-ILD, they should receive anticoagulation therapy at prophylactic doses if the risk of bleeding is low [57]. Other approaches are also possible. A recent case report revealed a possible beneficial effect of inhaled treprostinil for exacerbation of ILD that was refractory to conventional treatment [58]. However, the authors highlight the need for more scientifically rigorous studies in these settings. A recent observational study describes a potential therapeutic effect of intravenous immunoglobulins used during AE-ILD, supporting their use based on their anti-inflammatory and immunomodulatory mechanisms, as well as their favorable tolerability and safety profile [59]. Finally, palliative care of dyspnea is essential in AE-ILD, and some authors suggest that opiates can control dyspnea and should therefore be started alongside treatment of AE-ILD [60]. Further studies are necessary to determine the exact point at which to initiate opiates, the dose to be administered, and the duration of therapy. Palliative care should be provided through a multidisciplinary approach, incorporating measures such as rehabilitation, management of respiratory symptoms, anxiety, and depression, as well as effective communication with both patients and their caregivers. In addition, palliative care should be integrated into the management programs for respiratory diseases associated with a potentially poor prognosis, thereby improving quality of life through the assessment of physical and psychological needs and, ultimately, ensuring a dignified death [61].

## 4. Strengths and Limitations

Currently, the available information regarding AE-ILD remains limited. Therefore, we consider it a strength to have developed a simple diagnostic algorithm that incorporates variables applicable to routine clinical practice, such as blood tests, microbiological assessments, and radiologic findings. Through this approach, we aim to exclude conditions that complicate the differential diagnosis and to promote adherence to the current criteria used to define AE-ILD. Regarding treatment, to date, we believe this is the first therapeutic algorithm that summarizes all available options and provides a step-by-step protocol to follow in cases of refractory AE. The authors acknowledge the limitations of our study and of the proposed algorithms. First, both algorithms will require future validation through more rigorous scientific studies. Second, most of the treatments used in this area are not supported by randomized clinical trials and are based instead on observational studies, case reports, and the clinical experience of physicians who manage this condition daily. Finally, given the severity associated with AE-ILD, clinicians are often compelled to use certain therapies despite the aforementioned limitations, making it essential to follow a structured set of recommendations that allows for continuous reassessment of the condition while new therapeutic alternatives are investigated.

## 5. Conclusions and Future Directions

**Figure 3:**
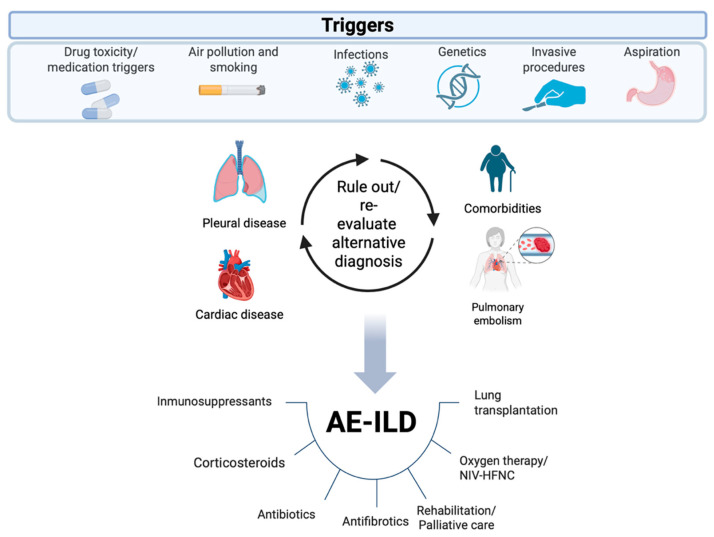
Potential triggers and management of acute exacerbation of interstitial lung disease. AE-ILD: acute exacerbation of interstitial lung disease; NIV: noninvasive ventilation; HFNC: high-flow nasal cannula.

At present, AE-ILD remains a life-threatening condition characterized by rapid and progressive clinical deterioration with a high associated mortality rate. Despite advances in diagnostic methods and therapeutic strategies, the pathogenesis of AE-ILD is still not fully understood. Therefore, early diagnosis and the exclusion of other conditions that may present with similar clinical features are essential. The algorithm developed in our study may serve as a useful tool in these scenarios. With regard to treatment, corticosteroids, immunosuppressive agents, antibiotics, and oxygen therapy continue to be used despite the lack of supporting clinical trials. Our review proposes a treatment algorithm for AE-ILD based on the best currently available evidence. To date, it is the first algorithm that describes step-by-step therapeutic options and specifies management strategies for refractory AE-ILD. We acknowledge that both algorithms will require future validation through more rigorous scientific studies. Going forward, there is a need for solid scientific evidence and clinical trials that enable appropriate, efficacious, and safe pharmacologic treatments. Clinical practice guidelines for the diagnosis and management of AE-ILD are urgently required. In the future, it will be essential to promote research projects that help elucidate the pathogenic mechanisms of this condition, enhance our understanding, and thereby enable the development of new therapeutic targets (Figure 3).

## Data Availability

The original contributions presented in this study are included in the article. Further inquiries can be directed to the corresponding author.
